# Navigating the dual dilemma between lives, rights and livelihoods: COVID-19 responses in China, Singapore, and South Korea

**DOI:** 10.1007/s12286-023-00555-x

**Published:** 2023-02-06

**Authors:** Heike Holbig

**Affiliations:** Goethe University, Frankfurt/Main, Germany

**Keywords:** China, COVID-19, Longitudinal perspective, Pandemic response strategies, Regime types, Singapore, South Korea, China, COVID-19, Längsschnittperspektive, Regimetypen, Strategien der Pandemiebekämpfung, Singapur, Südkorea

## Abstract

The COVID-19 pandemic has created a dual dilemma for governments worldwide: between the protection of lives and of individual rights, and more long-term between safeguarding lives and preserving livelihoods. Taking a dynamic approach, this paper asks how different regime types have navigated this dual dilemma by adjusting their pandemic-response strategies over the course of time. Three case studies from East Asia are selected to represent different regime types—autocratic China, hybrid Singapore, and democratic South Korea—that share experience with previous coronavirus episodes. Comparing the three cases between late 2019 to mid-2022, remarkable differences are found in the adaptability of response strategies. China’s authoritarian regime appeared to be at a clear strategic advantage due to its indifference toward individual rights during the first COVID-19 wave. In the longer run, however, the picture has changed substantially. While China has exclusively prioritized the protection of lives, fixating on its “Zero-COVID” strategy, Singapore has attached at least equal weight to sustaining livelihoods, experiencing a drawn-out zigzagging before pivoting to a “Living with COVID” strategy. Among the three cases, only South Korea has made consistent efforts to protecting individual rights while gradually recalibrating lives and livelihoods. Over time, the high degree of responsiveness of South Korea’s democratic regime has allowed for a relatively smooth transition to coexisting with the virus. The paper concludes with some lessons that European democracies might learn from pandemic responses in East Asia in a longitudinal perspective.

## Abstract (de)

Die COVID-19-Pandemie hat für Regierungen weltweit ein zweifaches Dilemma geschaffen: zum einen zwischen dem Schutz des Lebens und dem Schutz individueller Rechte, zum anderen längerfristig zwischen Lebenserhalt und der Sicherung wirtschaftlicher Existenzgrundlagen. Mithilfe eines dynamischen Ansatzes fragt dieser Beitrag danach, wie verschiedene Regimetypen durch Anpassungen ihrer Strategien zur Pandemiebekämpfung an sich verändernde Kontextbedingungen mit diesem doppelten Dilemma umgegangen sind. Untersucht wird diese Frage anhand dreier Fallstudien aus Ostasien – der Autokratie China, des hybriden Regimes Singapurs und der Demokratie Südkorea –, denen prägende Erfahrungen mit früheren Coronavirus-Epidemien gemeinsam sind. Ein Vergleich der drei Fälle im Zeitraum zwischen Ende 2019 und Mitte 2022 zeigt bemerkenswerte Unterschiede strategischer Anpassungsfähigkeit bei der Pandemiebekämpfung auf. Während der ersten COVID-19-Welle schien das autoritäre Regime Chinas klar im Vorteil, da es sich leicht über individuelle Rechte hinwegsetzen konnte. Mit der Zeit aber hat sich das Bild grundlegend gewandelt. Während China durchweg absolute Priorität auf den Schutz von Leben gesetzt und sich mit seiner Fixierung auf eine Null-COVID-Strategie in eine Sackgasse manövriert hat, hat Singapur die wirtschaftliche Existenzsicherung mindestens gleich stark gewichtet und damit einen ausgeprägten Schlingerkurs in Kauf genommen hat. Unter den drei untersuchten Fällen hat einzig Südkorea nachhaltige Anstrengungen zum Schutz individueller Rechte unternommen, während im Verhältnis zwischen Lebens- und Existenzsicherung mehrere strategische Anpassungen vorgenommen wurden. Im Zeitverlauf hat der hohe Grad an Responsivität der südkoreanischen Demokratie dazu verholfen, den Übergang zu einer Koexistenz mit dem Virus relativ reibungslos zu gestalten. Abschließend benennt der Beitrag einige Lehren, die europäische Demokratien in einer längerfristigen Perspektive aus den ostasiatischen Erfahrungen ziehen könnten.

## Introduction

When the SARS-CoV‑2 virus hit Wuhan in late 2019 and developed from an epidemic into a pandemic within three months, governments worldwide had to cope with an unprecedented health emergency. From a Comparative Politics perspective, the COVID-19 pandemic has been regarded as a kind of “natural experiment” (Capano et al. 2020, p. 285): governments across the globe have been facing the same challenge almost simultaneously but responded with different policies and varying effectiveness, thereby offering rich opportunities for comparison. Decision-makers on the ground vied with each other over how best to protect their citizens. As the pandemic unfolded with unprecedented speed, they had to rely on previous experience, existing capacities, and institutional routines—or trial and error given the lack thereof.

After weeks of criticizing China’s initial covering up of the epidemic and the draconian control measures in Wuhan, international media shifted to more self-critical narratives that unfavorably contrasted the pandemic response performance in Western democracies with that of autocratic China. By March 2020, when the virus haunted Europe with exponential infection rates, the Chinese party regime had successfully contained the first wave of infection. The building of two emergency hospitals in Wuhan within 11 days became an iconic media event, and massive lockdowns of in Hubei Province brought new infections to a halt within two and a half months. On March 19, the day Italy’s COVID-19 death toll surpassed the official figure in China and the virus had just started to rage in the United States, a *New York Times* article questioned whether this was “the price of an open society.” Statistical bias notwithstanding, the infection rates published by Johns Hopkins University painted an increasingly grim picture. The official death toll in China remained steady at below 4000, while US citizens had to mourn more than 100,000 COVID-19 victims by late May. By mid-2020, international media and social scientists alike increasingly suggested that democracies—and liberal-democratic regimes in the West in particular—had underperformed in terms of protecting their citizens’ lives and health.

More than two years later, by mid-2022, we see a much more mixed picture. Emerging virus variants have caused repeated waves of infection worldwide, leaving more than six and a half million people dead at the time of writing (with a death toll of more than one and a half million in the US and two million in Europe). In particular, the Omicron variant has emerged as a game changer due to its increased contagiousness combined with lower fatality. Governments that had contained the virus most successfully by late 2021 have faced new exponential infection rates, and previously effective measures to control the spread of the virus could be maintained only at a growing expense. While China’s continued “Zero-COVID” strategy has come at tremendous cost to the domestic economy, most other countries in Asia-Pacific as well as Europe and North America have pivoted to coexisting with COVID-19 and offset the pandemic’s negative effect on their economic activity. Over time, the initial dilemma of lives versus rights has been complicated by a longer-term dilemma: that of lives versus livelihoods.

Given the dramatic shifts in perceptions of relative successes and failures in tackling COVID-19 since 2020, this paper asks how different regime types in East Asia—autocratic China, hybrid Singapore, and democratic South Korea—have navigated the dual dilemma of lives, rights, and livelihoods by adjusting their pandemic-response strategies over the course of time. The analysis thus focuses on the responsiveness of different regimes in adjusting their strategies over time, thereby allowing (or not) for a dynamic balancing of their policy responses to the various challenges encountered during the different phases of the pandemic. While the balancing of pandemic responses and degree of responsiveness over time are analytically separate dimensions, the case studies demonstrate that they are still closely connected.

The paper is structured as follows. Based on a discussion of the existing literature, the research question, conceptual framework, and research design are first elaborated. The evolution of pandemic-response strategies in China, Singapore, and South Korea are then analyzed case by case, before offering a comparative discussion of strategic adaptations to changing environments made (or not) and their respective outcomes with regard to saving lives, safeguarding rights, and preserving livelihoods across regime types. Based on the empirical findings, the conclusion offers some lessons that European democracies might learn from the East Asian experience.

## Comparing the evolution of COVID-19 responses across regimes: a longitudinal perspective

Since spring 2020, Social Science scholars in the fields of Public Administration, Public Management, and Comparative Politics have shared a rich and multifaceted literature on COVID-19 pandemic responses across the globe. While the majority of publications have looked at individual country cases, a few have undertaken comparisons across countries and regime types. If applying conceptual frameworks at all, existing comparative research has largely applied capacity-related concepts. Examples include the use of “state capacity” (e.g., Mao 2021; Croissant and Hellmann 2023), “policy capacity” (e.g., Capano et al. 2020; Woo 2020), or “political capacity” (Kavanagh and Singh 2020) to understand differences in pandemic responses between autocracies, democracies, and hybrid regimes. While these capacity-driven approaches allow for rather fine-grained comparisons based on objectifiable empirical data, they usually limit their analysis to a given and mostly static set of variables observed at the pandemic’s initial onset. Thereby, they tend to ignore how pandemic-response strategies have evolved over time.

To capture these dynamic aspects, this paper applies a longitudinal perspective to understand how policy responses have been adjusted over the course of the pandemic, and how strategic adaptations have varied across regime types. Among the few publications sharing such a dynamic approach, Laishram and Kumar (2021) showed that, compared with other regime types, democracies responded to the pandemic less swiftly and effectively due to institutional hurdles and public concerns about violating democratic liberties. Cheibub et al. (2020, p. 1) argued that: “Democracies reacted slower than autocracies to the specter of the pandemic […] where rights are entrenched, encroaching on them is difficult. Yet […] when the threat of death became sufficiently severe, many democracies resorted to the same measures.” As their research suggests, autocracies appeared to be at a strategic advantage initially due to their relative indifference vis-à-vis individual rights and freedoms. The picture became more complex over 2021, however, when repeated waves of more contagious virus variants spread across the globe. Extending the analysis into 2022, we find a growing corpus of Economics literature on the disruption of economic activities through protracted lockdowns, quarantine requirements, and travel restrictions. This more long-term dilemma between “lives versus livelihoods,” as coined in respective publications (e.g., Jain et al. 2022; Kok and Woo 2021), has superseded the dilemma between lives and rights as discussed in Comparative Politics.

This paper proposes to combine these two hitherto disparate analytical angles and thus compare how different regime types have navigated the dual dilemma between saving lives, protecting individual rights and freedoms, and securing livelihoods over the course of the pandemic. More precisely, it asks how far relevant actors in autocratic, hybrid, and democratic regimes respectively have adapted their pandemic-response strategies to changing environments, thereby recalibrating the goals of protecting lives, rights, and livelihoods over time. The timeframe covers the period from the outbreak of COVID-19 infections in late 2019 through mid-2022, when the Omicron variant came to pose new challenges to public-health systems worldwide.

The focus here is on three country cases representing different regimes types in East Asia: the People’s Republic of China (PRC) as an autocratic regime, the Republic of Singapore as a hybrid regime, and the Republic of Korea as a democratic regime.^1^ While they vary enormously in terms of population size, socioeconomic development, and economic models, there are sound reasons for selecting cases from this specific world region. First, as COVID-19 originated in the region, the three countries started to react to its outbreak earliest—that is, in late December 2019 and early January 2020. Second, as attested to by the existing comparative literature, all three cases could rely on high state capacities to deal with the initial health challenge emerging (e.g., Croissant and Hellmann 2023). Third, and most importantly, all three countries had experienced earlier coronavirus epidemics: the SARS (Severe Acute Respiratory Syndrome) virus that raged in China and Singapore in 2002/03 and the less known MERS (Middle Eastern Respiratory Syndrome) virus that hit South Korea in 2015 (An and Tang 2020). Against this backdrop, the three countries had learned lessons from previous health emergencies, and their public-health systems and societies at large were thus on similar levels of preparedness when COVID-19 struck. From a longitudinal perspective, the commonalities with regard to timing, state capacities, and levels of preparedness in China, Singapore, and South Korea allow for similar starting conditions when comparing the evolution of pandemic-response strategies across regime types over time.

To operationalize the three variables—lives, rights, and livelihoods—of concern here, various quantitative indicators are chosen. Taking into account the differences in population size, economic models, socioeconomic developments, social welfare systems etc. in China, Singapore, and South Korea, highly aggregated macro indicators are combined to reflect trends, shifts, and relative changes over time rather than absolute ones. For each of the three dependent variables, two complementary country-specific indicators are chosen: For the “saving lives” variable, data is examined for a) cumulative COVID-19 deaths per one million people and b) fatality rates (provided by Our World in Data). For the “safeguarding rights” variable, reliance is on a) the Pandemic Backsliding and Violation of Democratic Standards Indices as compiled by the V‑Dem team and b) the Bloomberg COVID Resilience Ranking, comprising data such as lockdown severity and mobility restrictions. For the “securing livelihoods” variable, we use International Monetary Fund (IMF) data about a) COVID-19 related fiscal stimuli as percentage of gross domestic product (GDP) and b) changes in real GDP growth rates. The case studies are based furthermore on the rich existing Social Science literature on pandemic responses in East Asia as well as on primary sources such as official policy documents, strategy papers, and regional media coverage.

The evolution of pandemic-response strategies in the three regimes will be traced case by case before then systematically comparing them. The main institutions and actors involved, their communication and interagency coordination, resource mobilization, the speed, scope, and intensity of response measures as well as their adaptations to changing contexts will all be examined. The analysis demonstrates that while all three regimes have come up with relatively effective pandemic responses in certain moments, their ability to adjust these strategies varies significantly in the longer term.

## Locked in seclusion: ideological fixation on Zero-COVID in the Chinese party-state

When physicians in Wuhan, the capital of Hubei Province, warned of a new type of pneumonia in December 2019, local party-state authorities reacted with a mixture of trepidation and nonchalance. As far as official communications on the ground during the first few weeks of the epidemic have been recorded, an epidemiological survey was conducted on December 29, followed by daily meetings of the local government from December 31 onward and the closure of a local wet market regarded as hotspot on January 2, 2020. The priority of municipal and provincial authorities, however, was to maintain public order in a politically important period. The annual “two sessions”—meetings of the People’s Congress and the People’s Political Consultative Conference at the municipal and provincial levels—had been scheduled for January 6–17, to be finished in time before the lunar spring festival starting on January 25. In light of the SARS-CoV‑1 pandemic experienced in 2002/03 in the same region, with a death toll of more than 600, news of another coronavirus-type infection was perceived as highly sensitive. In line with long-standing rules of the party’s propaganda apparatus, whose priority was to prevent the outbreak of social panic, physicians in Wuhan were instructed not to spread “rumors” about the infection, and state media were reminded not to report about the novel pneumonia on their own initiative before being licensed to do so in accordance with centralized news reporting (Holbig 2020). In this initial information vacuum created over three weeks by the sensitivities of an authoritarian party-state, social media spread hearsay about growing numbers of infections and hospitals working beyond their limits, while neighboring regions and countries such as Hong Kong, Taiwan, or Singapore started to screen passengers from Wuhan.

Meanwhile, actors at the central level appeared torn between being on high alert and inertia. According to official sources, the National Health Commission sent an expert team to Wuhan on December 31, 2019, while the National Center for Disease Control informed the World Health Organization (WHO) about the new virus on January 3, 2020, and succeeded in isolating the first novel coronavirus strain four days later (SCIO 2020, pp. 4–6). Despite rapidly increasing infection rates in Wuhan, however, it maintained the official line that “no solid evidence has been found to prove person-to-person transmission” (Mei 2020, p. 314). This ambiguous assessment was revised only on January 20 when chief epidemiologist Zhong Nanshan officially verified contagion between humans. The obvious lack of coordination between local and central authorities has been explained as a typical outcome of China’s “fragmented authoritarianism,” meaning the quasi-federalist governance structures in place after the devolution of administrative powers to lower levels over the course of decades (Mei 2020). This explanation, however, ignores the information paradox typical of authoritarian party regimes, where responsible actors collude to prevent social panic rather than collaborating to tackle a given health emergency early on. The result is a kind of misdirected alertness which tends to disincentivize or even paralyze local initiative (Holbig 2023, pp. 247–250).

The pandemic response changed dramatically just before the Chinese spring festival. At a time when millions of Wuhan citizens left the city to celebrate the lunar new year with their families, the central party-state leadership eventually took over the reins and switched to national-crisis mode. While paramount leader Xi Jinping had previously remained in the background, he was now celebrated in the state media as resolute mastermind of the country’s emergency response, personally addressing the “people’s masses” and vowing to “put the safety of their lives and their physical health first” (*People’s Daily*, January 21, 2022). A hierarchical command structure was set up in Beijing under the party’s auspices, and thus unimpeded by legislative procedures reserved for state organs, to orchestrate a panoply of top-down response measures. Within days, the party established a “Central Leading Group for Epidemic Prevention and Control” chaired by Premier Li Keqiang (and with the party’s propaganda and ideology czar Wang Huning as vice-chair) and a “Joint Mechanism of the State Council for Pneumonia Epidemic Prevention and Control” tasked with the coordination of epidemic-response work across 32 departments. The latter’s head, Vice-Premier Sun Chunlan, was then sent to Wuhan to personally supervise the lockdown measures in Hubei Province (SCIO 2020, p. 13). From January 25 onward, Wuhan and other nearby metropolises were sealed off, strict curfews implemented and patients with symptoms isolated, travel bans within China and to foreign countries imposed, as well as mass testing and digital tools applied. In the absence of legal hurdles preventing misuse of personal information, CCTV cameras, drones, and GPS data from mobile-phone operators were used to monitor peoples’ movements, and contact-tracing apps were provided via social media and e‑payment applications (Duchatel et al. 2020, p. 50–52). Based on a cross-provincial collaboration mechanism that coordinated the provision of medical staff and equipment to the sealed-off cities in Hubei Province from 16 other provinces as well as on support from local mass organizations, the party-state established a huge logistics network to maintain a 76-day lockdown in a region populated by tens of millions of people (Mei 2020, pp. 315–16).

The impressive capacities of China’s authoritarian party regime to mobilize massive resources within a short period of time were seen also in other policy fields. Under the guidance of the Central Leading Group, the Ministry of Industry and Information Technology ordered factories to step up the production of critical medical equipment such as masks, testing kits, protective suits, and infrared body thermometers, guaranteeing to purchase and stockpile overcapacities (Duchatel et al. 2020, p. 48). Similar measures were taken to develop domestic vaccines: already by late 2020, China approved its first own inactivated anti-COVID-19 vaccine, being followed by six more domestic vaccines by late 2021 (Ye 2022). This allowed the PRC to inoculate hospital and other critical infrastructure personnel early on, but also to donate its vaccines to developing countries within the COVAX scheme—an international effort to deliver vaccines to poorer countries on an equitable basis, after approval by the WHO. Last but not least, financial subsidies were granted to enterprises and households suffering from the lockdowns, while domestic production was stepped up in other critical industries to reduce world-market dependencies.

Assured by their successful response to the first COVID-19 wave in Wuhan, party-state authorities continued their zero-tolerance approach to ferret out new infections immediately also in subsequent waves that Beijing and various other parts of the country would experience over the next two years. The standard response repertoire consisted of mass testing, close-meshed contact tracing, strict confinement and rigorous lockdowns—the latter sometimes of tens of million people at a time, aiming to bring infection rates down to zero within a couple of weeks (Huang 2022). While this Zero-COVID strategy appeared effective in the sense of stamping out sporadic outbreaks and limiting the official death toll to less than 5000 before December 2021, it came at increasing economic and social costs. Given the size and growth potential of the Chinese economy, however, the overall fiscal burden resulting from various local lockdown episodes still seemed moderate relative to total GDP. Compared to the heavy economic impact that the pandemic caused in other world regions, the PRC was among the very few countries worldwide to end the year 2020 with a positive GDP growth rate of 2.2%.

As this account suggests, the apparent initial effectiveness of China’s pandemic response can be attributed to its strong coercive, extractive, and administrative capacities as an autocratic regime. This was supported by the strong ideological cohesion that the communist party-state could mobilize once it had switched to national-crisis mode. Yet, with the much more contagious Omicron variant entering the scene in late 2021, the previous effectiveness of the country’s Zero-COVID strategy turned into a twin liability. On the one hand, the party-state leadership has wedded itself to an ideological contest with the US, arguing that the success of its Zero-COVID approach epitomizes the superiority of China’s socialist system vis-à-vis Western democracies. The regime’s claim to protect citizens lives at all costs, underlined by Xi Jinping’s heroic role as commander-in-chief of the nation’s war against COVID-19, has cultivated not only acute anxieties related to the virus but also high expectations among the populace of continued protection from the virus (Holbig 2020). On the other hand, the instant effectiveness of China’s Zero-COVID policy appears to have reduced the incentives to pursue a systematic vaccination program.

As US health expert Huang Yanzhong (2022) has argued, despite the early availability of various conventionally produced domestic vaccines and a notable vaccination rate of 83% by January 2022, the Chinese population lacks adequate immunity to face the more contagious Omicron and other emerging variants with confidence. One reason for the significant immunity gap is the relatively low efficiency of China’s inactivated vaccines compared to mRNA vaccines developed in the US and Europe, which have been disparaged in the country’s official propaganda in line with its broader anti-Western narratives. Another reason is the prioritization of younger generations—healthcare and service-sector workers in particular—in vaccine rollouts, leading to relatively low vaccination rates among the elderly despite them being disproportionately more vulnerable to all COVID-19 variants. “Unless China abandons the zero-COVID mentality,” Huang argued in January 2022, “it may have to adopt ever more draconian controls until the virus completely disappears or face an Omicron tsunami of almost unimaginable proportions” (Huang 2022).

Since then, the Omicron wave has reached Shanghai, the country’s economic powerhouse and export hub, which was locked down according to the same rigorous playbook. With more than two months of repeatedly extended confinement, public resentment among Shanghai’s middle-class urbanites appeared to reach a tipping point (Zhuang 2022). Since then, similar patterns have been observed in various other metropolises across China, where citizens have taken to both online and offline forms of protest to vent their anger about extended confinement, a lack of food and daily necessities, and even losses of life due to neglect, accidents during forced quarantine, or from other unrelated diseases in a health system preoccupied with Zero-COVID.^2^ In the meantime, news has emerged that Chinese state-owned enterprises are experimenting with domestic mRNA vaccines—but there still seems a long way to go before they can be rolled out and bring down hospitalization rates (Ye 2022).

Yet, given the autocratic leadership’s ideological fixation on Zero-COVID, an exit from this strategy—which would imply a personal criticism of Xi’s leadership—appears increasingly difficult. Instead, a 33-point “stabilization” scheme launched by the State Council in June 2022 later combined increased tax rebates for retail, agriculture, food, services, education, health, and entertainment industries, fast-track infrastructure projects, car-purchase incentives, financial support for small and medium-sized businesses and for foreign-invested enterprises, as well as increased unemployment insurance (Liu 2022). With this new scheme in place to offset the economic impact of Zero-COVID, it seems highly unlikely that China’s authoritarian party regime will transition to a strategy of coexisting with the virus anytime soon. Locked in national-crisis mode, the mobilization of anti-Western sentiment at home and the country’s isolation from global interaction seem to reinforce each other, increasingly overriding concerns about economic performance and people’s wellbeing. Despite strong state capacities, an adjustment of China’s pandemic-response strategy seems increasingly difficult.

## Zigzagging through the pandemic: Singapore’s difficult choice between lives and livelihoods

Compared to the initial delay and ambiguity of China’s reactions to the SARS-CoV‑2 outbreak, the Singaporean city-state made up of 5.7 million people reacted most quickly to the news from Wuhan. Already on January 2, 2020—three weeks before the first COVID-19 case would be identified—the Ministry of Health requested doctors report patients with pneumonia-like symptoms and set up temperature checks for incoming passengers from Hubei Province. In a situation of general uncertainty, the Singapore government could fall back on a complete set of institutional safeguards and emergency routines that had been established in the wake of the SARS epidemic costing 33 lives back in in 2002/03. Since then, regular updates of emergency plans, capacity replenishments, and government stockpiling of masks, personal protective equipment, pharmaceutics, and similar had ensured high levels of preparedness. Among other institutions created in the wake of the SARS crisis, the Ministry of Health oversees so-called Public Health Preparedness Clinics for quarantine purposes at the neighborhood level, a National Public Health Laboratory, as well as a National Centre for Infectious Diseases that was officially opened in September 2019—meaning just a few months before the COVID-19 outbreak. The new facility with a capacity of 330 isolation beds was designed to manage an outbreak on the scale of SARS (Kurohi 2019).

Facing the new COVID-19 virus, the Ministry of Health capitalized on its healthcare capacities and emergency routines within the space of just a few weeks between early January and mid-February 2020. On February 8, Prime Minister Lee Hsien Loong officially addressed Singaporeans to explain the situation, reduce anxiety, and instill a “sense of normalcy” among citizens who had started panic buying, while purchase limits were imposed on supermarket chains (Phua 2020). Border controls and travel restrictions for short-term visitors were stepped up successively to reduce the risk of imported cases. Also, in collaboration with hotels and private enterprises, the government used CCTV footage to track cases, and developed new smartphone applications based on smart-city data to record and trace infected persons in close proximity and allow health administrators to contact at-risk persons directly. While the use of the Bluetooth-based “TraceTogether” app was voluntary, the smart “SafeEntry” system was designed as a compulsory digital check-in and check-out tool for public spaces such as offices, schools, universities, and malls. A few academic voices skeptical of such surveillance technologies would be heard, but in the name of collectively combating the COVID-19 enemy such concerns about data privacy and individual freedoms were set aside in the public discourse (Das and Zhang 2020). Equipped with these intrusive digital tools of contact tracing and cluster identification, strict confinement policies, and heavy fines and jail sentences to deter violations of quarantine rules, the city-state was able to limit the spread of COVID-19 to a few isolated cases and to avoid a general lockdown during the first quarter of 2020 (Duchatel et al. 2020, pp. 93–95). At the time, Harvard epidemiologists considered “Singapore to be a gold standard of near-perfect detection” (Niehus et al. 2020, p. 3).

The situation changed in late March 2020 however, when infections suddenly surged among Singapore’s migrant workers living in densely packed dormitories on the city’s fringes. Obviously, this vulnerable group—which constitutes more than one-third of the city’s workforce, mostly in the service and construction industries—had flown under the radar of an otherwise well-prepared healthcare system. The exponential increase of COVID-19 infections in migrant dormitories exposed the government’s blind spot in dramatic ways, causing a “pandemic of inequality” (Tan 2020a) in the affluent city-state.

**Fig. 1:**
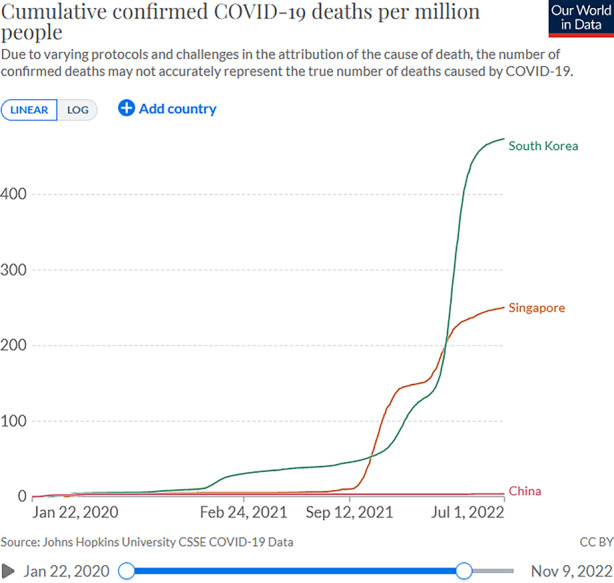
Cumulative confirmed COVID-19 deaths per million people in China, Singapore, South Korea. *Source*: Ritchie et al. (2020)

This blind spot appears characteristic of a hybrid regime featuring formal electoral institutions and competition from smaller opposition parties but largely disregarding the voices of non-state actors and bottom-up initiatives to collaborate with civil society. In fact, civic organizations such as Transient Workers Count Too (TWC2) had advocated for migrant workers’ rights over two decades and repeatedly pointed out their desolate living conditions, but their warnings had been consistently ignored (Woo 2020, pp. 354–55). To tackle the exponential increase of dormitory cases, the government used its discretionary powers to institute a “circuit-breaker” lockdown in early April, closing all businesses except for essential services and shifting to home office and home schooling for one month. Three weeks later, infection rates declined and the second wave had flattened out by June 2020 (Woo 2020), with a very low death toll (Fig. 1).

To reduce the socioeconomic impact of the pandemic, the government also decided on massive fiscal stimuli. Between February and May 2020 alone, four consecutive rounds of capital injections were approved to stabilize the city’s economy. Compared to other Asian countries, Singapore’s fiscal measures as a share of its 2020 GDP ranked second only to Japan (Kirkegaard 2021). When general elections were held according to schedule in early July, they served as a de facto referendum on the government’s pandemic response. While the ruling People’s Action Party (PAP) under Lee Hsien Loong’s leadership secured its 15th consecutive term in government since 1959, the main opposition Workers’ Party, which had criticized the poor handling of the COVID-19 clusters in migrant-worker dormitories, won a record high of 10 out of 93 seats in parliament. Despite the previous fiscal-stimulus packages, Prime Minister Lee confessed that the government had not done enough to ease the uncertainties Singaporeans faced during the “crisis of a generation” (Tan 2020b). According to election analysts, the pandemic had demonstrated the diminishing effect of social transfers to the population and to businesses Singaporeans had been used to for decades (Guimaraes 2020). At least temporarily, the pandemic jeopardized the hybrid regime’s long-standing social contract based on material wealth in exchange for compliance with the PAP’s paternalist leadership.

As one of the world’s most vibrant international trade and service hubs, Singapore’s wealth is highly dependent on the global market, creating a particularly sharp dilemma between protecting people’s lives and sustaining livelihoods here. Aware of this conundrum, under the leadership of Lee the government presented another fiscal stimulus in August 2020 and an even more sizeable “COVID-19 Resilience Package” in February 2021. The latter was explicitly designed to “safeguarding public health and re-opening safely” (SG Gov 2021), signaling the political will to end the rigid scheme of travel bans. Besides substantial allocations to public health and safe reopening measures, the package foresaw recovery grants for workers and businesses as well as subsidies for the worst-hit sectors such as aviation, transport, arts, culture, and sports. Having learned lessons from the previous spike of dormitory cases, enterprises with high shares of migrant workers received extra benefits to ensure their livelihoods and adequate healthcare provision. At the same time, the “Singapore United Jobs & Skills” program created 15,000 new openings in the public sector alone and offered extensive training opportunities, including for citizens over the age of 40—possibly also with a view to reducing dependency on foreign workers (Ho 2020).

The government’s cautious attempts to loosen travel restrictions and to “safely reopen” the economy were, however, torpedoed by the Delta variant, which would come to dominate from May 2021 onward. A public controversy about the government’s pandemic-response strategy was sparked when Calvin Cheng, an ex-member of parliament, derided Singaporeans as a “the biggest nation of crybabies in the world” who “have lived in Disneyland for too long […] and have a warped perspective of real life” (Teh 2021). While ideas to create a “travel bubble” between Singapore and Australia had been aired for months, the scheme did not manifest before late 2021. Facing the mounting economic costs of sustained mobility restrictions, Lee decided in early October to prepare the public for a transition to “Living with COVID” (Lee 2021). The Ministry of Health stepped up its vaccination program to increase Singapore’s inoculation rate, which already had been among the highest in the world, by offering Pfizer-BioNTech and Moderna vaccines for free and other vaccines for self-payers who did not trust mRNA-based ones (Chiu and Chin 2021). In late October, booster vaccinations were rolled out for all residents above the age of 30, and in December 2021 the Ministry of Health—stopping just short of introducing a mandatory vaccination scheme—began charging COVID-19 patients who were “unvaccinated by choice” (SG MoH 2021).

Criticized for its “messy” and “rocky” reopening strategy (McGregor 2021), the government hesitated again in the face of the Omicron variant in December 2021 but eventually returned to gradually loosening travel restrictions and border controls after its peak had been reached in February 2022. At the time, the Ministry of Health, in one of its regular COVID-19 updates, noted that: “As Singapore’s incidence rate is now comparable with most overseas destinations, imported cases are unlikely to affect the trajectory of local cases” (SG MoH 2022). Even with new Omicron waves in May and June 2022, the government remained committed to Living with COVID, trying to strike a balance between maintaining an open economy and monitoring hospitalization and mortality rates to keep infection levels manageable.

As the case of Singapore’s hybrid regime shows, strong state capacities and high levels of preparedness allowed for a swift and resolute response immediately after the outbreak of COVID-19, while individual rights and privacy concerns were generally neglected in the name of a collective fight against SARS-CoV‑2. A first setback happened when infections surged among Singapore’s marginalized migrant workers, but the government tackled this blind spot by subsequently including this vulnerable group in its extensive fiscal-stimuli programs and COVID-19 resilience packages. Given Singapore’s reputation of effective governance by a highly professional civil service, however, the transition to Living with COVID appears to be a surprisingly difficult and drawn-out process that continues apace at the time of writing. Through 2021 and into 2022, concerns over the city-state’s economic performance and people’s livelihoods have weighed more heavily compared to the protection of lives at all costs. Once this hard lesson had been learned, incidence rates were “normalized” at average international levels, while the professional public-health system has allowed infections to be kept at manageable levels so far.

## Keeping abreast: balancing lives, rights, and livelihoods in South Korea

During the initial phase of the COVID-19 epidemic, South Korea’s response was similar to Singapore’s. Already from January 3, 2020, onward, the government introduced screening measures for travelers from Wuhan as well as strict quarantine policies that were stepped up over time. As in Singapore, the government was able to rely on a well-prepared public-health and disaster-management system that had been institutionalized in the wake of the MERS epidemic, a Middle Eastern coronavirus variant that had cost the lives of 38 South Korean citizens in 2015. In particular, the Korea Centers for Disease Control and Prevention (KCDC), which had been established after the SARS epidemic in 2003, was granted extended authority for interministerial coordination in case of health emergencies in 2015. In the same year, South Korea’s Infections Disease Control and Prevention Act was amended to provide the Ministry of Health and Welfare and the KCDC with legal authority to collect citizens’ private data without court approval or personal consent (Kim 2020a). Regular updates of emergency plans and frequent emergency drills were designed to ensure readiness of communication and crisis-management procedures. In December 2020, just before news emerged about a novel coronavirus in Wuhan, the KCDC had launched a “wargame” desktop exercise among its experts to prepare for an unforeseen disease outbreak (Shin 2020).

Based on the KCDC’s preemptive alertness and institutional routines, the Ministry of Health and Welfare was able to react to the real-world outbreak of SARS-CoV‑2 in a highly agile manner, thus avoiding a major contagion for six weeks. At the same time, the well-rehearsed epidemic-response procedures allowed the government to remain calm. Despite a petition demanding a general travel ban on all incoming Chinese visitors that was signed by more than half a million South Korean citizens through late January, the government still adhered to its cautious policy of targeted border controls (Duchatel et al. 2020, p. 100). Instead, a Central Incidence Management System for the Novel Coronavirus as well as a Pan-Government Countermeasures Support Headquarter headed by the Minister of Interior and Safety were established in late January, signaling a first shift of taskforce leadership. The swift upgrading of taskforce structures allowed the KCDC, the Ministry of Health and Welfare, and the Ministry of Interior and Safety to coordinate pandemic response measures such as health checks for incoming travelers, testing, and quarantine efforts—not only across relevant ministries but also administrative levels too. Besides the prime minister, his deputies, and all ministers, representatives of local city and district governments attended the pan-governmental format as well, meeting two to three times a week (Dyer 2021).

Another strength of the South Korean government was its close collaboration with private enterprises to speed up the domestic development of testing kits early on. The KCDC granted fast-track approval to a first kit developed by Kogene Biotech on February 4; three days later, a first batch of tests was delivered to 50 clinics, allowing health authorities to test hundreds of thousands of citizens within a few days. After other Korean companies had taken up test production, the country was able to export some of its capacities to Europe and other world regions (Duchatel et al. 2020, pp. 102–103). In light of this early success, the WHO and various international experts recommended South Korea as a policy-response model in the field of mass testing (You 2020).

The situation changed dramatically in mid-February, however, when a major outbreak occurred in the southern city of Daegu. Infections increased exponentially after a female superspreader with contacts to Wuhan infected hundreds of members of the Shincheonji Church, a protestant religious group, during mass service. After sending a special taskforce and a rapid response team to Daegu to investigate the Shincheonji case in the face of strong resistance from Church members, KCDC eventually received the contact data of 210,000 members. In the following weeks, central and local health authorities collaborated closely to monitor this target group. Drive- and walk-through test centers became another early innovation in the pandemic. Shincheonji members were also provided extra face masks, a rationed item in South Korea at the time, and granted financial subsidies in case of infection or quarantine (Duchatel et al. 2020, pp. 101–103; Kim 2020b).

The Daegu outbreak resulted in a first adjustment of the government’s pandemic-response strategy. While the focus so far had been on test and trace, it was now complemented by a containment and mitigation strategy that aimed to prevent larger community spreads of the virus by strictly isolating and treating confirmed cases. On February 23, the government announced the highest alert level and established a Central Disaster and Safety Countermeasures Headquarters, headed by President Moon Jae-in himself, to function as a command center for all pandemic-response measures requiring coordination between central- and local-level agencies (SK MoH 2020). On March 15, in an unprecedented move, Moon declared the city of Daegu a “special disaster zone,” thereby scaling up disaster-management facilities in the region. In due course, the government suspended religious gatherings, sports events, and closed entertainment facilities as part of a two-week “social-distancing campaign,” while also increasing fines and jail sentences for quarantine violators (Duchatel et al. 2020, p. 99).

Demonstrating its respect for individual rights and freedoms, the government collaborated with various public and private agencies to develop a set of highly sophisticated digital applications for contact tracing, which formed another important element in the government’s pandemic-response toolkit. Initially, a smartphone-based “self-quarantine safety protection” app was introduced to trace and tag infected persons and close contacts, which allowed public-health workers to monitor symptoms via daily phone calls and alerted them when quarantine rules were violated. Later on, a tracking system was developed that immediately collected big data such as mobile-phone location, credit-card-usage history, CCTV footage, and other large-scale urban information. The government repeatedly assured citizens about protecting their private data as far as possible, by guaranteeing anonymity or applying strict time limits on the storage of sensitive data (Kim 2020b, pp. 5–6). While names were not revealed, KCDC lists of confirmed cases, which were published online and relayed through local and social media, would include information such as age, sex, neighborhood, occupation, employer, and travel history. After public criticism by a coalition of 17 human rights groups and an intervention by the National Human Rights Commission, the KCDC updated its guidelines to prevent information leaks that might allow patients to be inadvertently identified (Duchatel et al. 2020, pp. 104–105).

Contrasting with the intrusive tracing tools employed in China and Singapore, the democratic government of South Korea proactively made explicit and continuous efforts to balance the protection of lives with the respecting of individual rights and liberties. As the Daegu episode showed, effective epidemic control was prioritized with the help of big data-based digital tools when deemed necessary though.

**Fig. 2:**
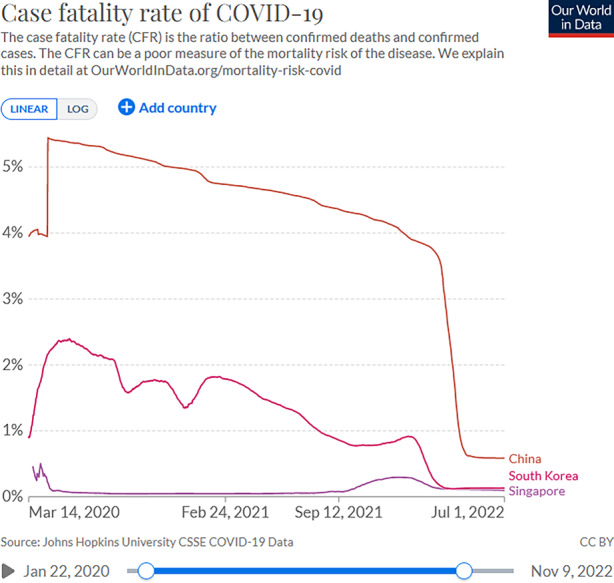
COVID-19 case fatality rates in China, Singapore, South Korea, *Source*: Ritchie et al. (2020)

With these swiftly evolving policies, the death toll could be limited to below 1000 over the first 12 months of the pandemic, despite relatively high infection rates (Fig. 1 and 2). While South Korean numbers clearly exceeded those in China and Singapore, one should bear in mind that European democracies of comparable population size had lost many times more lives during the same period. One of the reasons for the relatively low fatality rate in South Korea might be found in the close cooperation between state and private healthcare facilities, which allowed doctors to classify and treat confirmed cases according to the severity of people’s symptoms. While confirmed cases were isolated in community centers, patients with mild symptoms were treated in smaller, mostly private clinics; those with moderate or severe symptoms, conversely, were immediately hospitalized in national infectious disease hospitals, thereby also ensuring regular hospitals’ continued ability to handle non-COVID-19 patients. Moreover, medical diagnosis and therapies benefited from innovative Artificial Intelligence technology (Kim 2020b). Other positive factors emphasized in the literature include high levels of awareness and compliance among Korean citizens thanks to transparent, consistent, and credible information provided by the government (Moon 2020), and a synergetic collaboration between the state, experts, and civil society (Mao 2021). Contrasting with the top-down orchestration of pandemic responses in China’s authoritarian and Singapore’s hybrid regime, the transparency and collaborative endeavors of South Korea’s democratic government clearly benefited the efficiency of its COVID-19 response in the longer term.

In addition, the South Korean government took various accompanying measures to offset the pandemic’s lasting economic impact. Similar to Singapore’s economic-response policies, various relief packages were provided to stabilize financial markets and employment, to support the self-employed, small and medium-sized enterprises, and other vulnerable businesses, as well as to increase household income and spur consumption (Dyer 2021, pp. 21–22). Compared to Singapore and other advanced economies in the region, however, the fiscal stimuli as part of GDP remained at a significantly lower level, being here more targeted and rolled out proportionately as the situation evolved (Kirkegaard 2021; Table 3). In July 2020, these short-term fiscal policies were complemented by a long-term initiative labeled the “Korean New Deal”—a national strategy to enhance the country’s economic transformation while strengthening the social safety net. With this initiative, the government envisaged massive public investment in South Korea’s digital economy (5G technology, automation, AI, machine learning, e‑commerce, tele-medicine, and similar) and green economy (transition to low-carbon and eco-friendly economy, green technologies, energy-efficient manufacturing, and the like) (SK MoEF 2020). A year later, in July 2021, the government announced an updated version, the “Korean New Deal 2.0,” which had been adjusted to cater to new demands in the fields of education, to widening socioeconomic gaps and growing related insecurities among young adults, and to increasing labor demands in new industries (SK MoEF 2021). As these initiatives show, instead of simply cushioning the negative impact of the COVID-19 crisis, recovery funds were steered toward promoting new technologies, creating new jobs and upskilling the workforce, as well as accelerating the transition to a more sustainable and competitive economic structure.

Meanwhile, in the interest of safeguarding individual liberties and livelihoods, South Korea chose not to shut down its economy and abstained from imposing general mobility restrictions and border closures. In consequence, infection rates have evolved much more dynamically compared to China and Singapore. Despite a delayed rollout of mRNA vaccines due, among other factors, to an early commitment to the COVAX initiative, the government was able to control the Delta variant that emerged in spring 2021 quite successfully (Dyer 2021). When the government announced a “Living with COVID” plan in late October of that year, however, envisaging a three-stage scheme to end all restrictions by February 2022, the Omicron variant forced it to slow down the scheme’s implementation, accelerate vaccination rollouts (including offering booster shots to young people from the age of 18), and introduce a vaccine pass (Kim 2021). Since November 2021, infection rates have increased steadily—with an exponential increase in spring 2022. As of mid-March 2022, the death toll had exceeded 10,000; halfway through that year it would stand at 25,000 meanwhile.

Despite rising infection rates, South Korea’s new president Yoon Suk-yeol, who was voted into office in regular elections in March 2022, continued the implementation of Living with COVID (albeit according to an adjusted time scheme), eventually pivoting to coexistence with the virus. It was one of the first countries in the region to do so. After various strategic adaptations to a changing environment, South Korea’s democracy has been able to keep its economy open and growing, ensure people’s livelihoods, safeguard individual rights, and to maintain a more or less “normal” social life while accepting moderate fatality rates.

## Rights, lives, livelihoods: how different regime types have navigated the dual dilemma

After separate analyses of pandemic response trajectories in China, Singapore, and South Korea, we can now compare how each respective regime has navigated the dual dilemma between lives, rights and livelihoods over the course of time. Most obviously, China’s autocratic party regime has prioritized the protection of lives above everything else throughout the analyzed time period. According to official statistics, among a population of 1.4 billion, the cumulative death toll stood at a remarkable low of four COVID-19-related deaths per one million people as of July 1, 2022, compared to 251 in Singapore and 474 in South Korea (Fig. 1). China compares less favorably when we look at case fatality rates however, meaning the ratio between confirmed deaths and confirmed cases. Although this ratio must be interpreted with caution, the high fatality rate here of between 4% and 5.4% recorded during the first two years (falling to 0.59% by mid-2022 thanks to the Omicron variant) contrasts markedly with a pre-Omicron peak of 0.5% (falling to 0.10% by mid-2022) in Singapore and of 2.4% (falling to 0.13% by mid-2022) in South Korea (Fig. 2). As these figures suggest, the effective protection of lives in China has been hampered not only by healthcare capacities but also by the massive spatio-temporal scope of lockdowns at the expense of taking individual circumstances into account. Singapore’s and South Korea’s advanced healthcare systems as well as individualized treatment depending on the severity of illness have clearly helped to bring fatality rates down to record lows, also from a global perspective.

Comparing the protection of rights across regime types, the overall picture is unsurprisingly clear. According to V‑Dem’s Pandemic Violations of Democratic Standards Index, which captures the situation between March 2020 and June 2021, major violations of individual rights and freedoms in terms of discriminatory measures, derogations from rights and restrictions of media freedom have been recorded in autocratic China, as have more moderate violations through the enforcement of lockdown and quarantine measures too. In Singapore’s hybrid regime, meanwhile, moderate violations have been observed in terms of discriminatory measures at the individual level (in particular, against migrant workers) and restrictions on media freedom, while no such violations were recorded in democratic South Korea (Table 1).

**Table 1:** Pandemic Violations of Democratic Standards in China, Singapore, South Korea

Country	Discriminatory Measures	Derogations from non-derogable rights	Abusive enforcement	Restrictions of media freedom
China	Major violations	Major violations	Moderate violations	Major violations
Singapore	Moderate violations	No violations	No violations	Moderate violations
South Korea	No violations	No violations	No violations	No violations

*Source*: V‑Dem Net (2020)

Please note: The Pandemic Violations of Democratic Standards Index (PanDem) covers the period from March 2020 to June 2021. It captures the extent to which state responses to Covid-19 violate democratic standards for emergency responses. Among the 7 Component Indicators included in the index, we selected 4 indicators which directly address violations of individual rights and democratic freedoms.

To complete the picture for the remainder of the period under study, Bloomberg’s COVID Resilience Ranking, which reflects the world’s major economies’ changing ability to cope with the virus over time, may give at least a clue here. Published monthly for the period November 2020 to June 2022, the lockdown severity score—measuring restrictions on individual mobility—is by far the highest for China; so are the disruptions to daily social and economic life, as reflected in massively reduced community mobility compared to pre-pandemic levels, resulting in a very low overall ranking (51 out of 53 countries worldwide). While the scores for Singapore are much more favorable, the COVID Resilience Ranking still records significant disruptions to people’s normal social and economic life as measured by the community mobility score for mid-2022. At this point in time, community mobility in South Korea has not only normalized but even exceeded pre-pandemic levels according to Bloomberg, which ranked the country’s overall resilience to cope with the virus first among the world’s major economies in July 2022 (Table 2).

**Table 2:** COVID Resilience Ranking for China, Singapore, South Korea, June 2022

Country	Lockdown severity (0–100 scale)	Community mobility (% change vs pre-pandemic baseline)	Resilience Score (0–100 scale)	Ranking among 53 major economies
China	79	−22.0	54.7	51
Singapore	32	−8.8	75.6	14
South Korea	14	+5.9	80.9	1

*Source*: Bloomberg (2022)

Please note: The Bloomberg COVID Resilience Ranking includes 53 countries representing the world’s major economies and has been updated monthly between November 2020 and June 2022. It includes 11 indicators that reflect the countries’ abilities to cope with the virus. Two of them, lockdown severity and community mobility, have been selected here as they directly bear on individual freedoms, i.e., restrictions to individual mobility and to social and economic activity. The data offered in the table reflect the situation of late June 2020.

This brings us to the third policy target: the protection of livelihoods. From a longitudinal perspective, this is the variable where changes become visible only in the longer term. To be able to compare the three cases despite their stark socioeconomic differences, we consulted two indicators published by the IMF to capture longer-term trends at a highly aggregated level. Looking at the proportions of individual countries’ fiscal measures taken in response to the COVID-19 pandemic and to stabilize the socioeconomic situation (measured as percentage of GDP), which are available via the IMF’s Fiscal Monitor Database for the period running from early 2020 to September 2021, we find a surprisingly low figure of 6.1% of GDP for China. Strikingly, Singapore’s fiscal measures, which amounted to 23.1% of GDP by fall 2021, ranked among the world’s highest. South Korea, which had earmarked 16.6% of its GDP for pandemic-response measures over the same period, occupied a medium rank (Table 3), thereby coming close to average percentages of fiscal stimuli observed in Western liberal democracies.

**Table 3:** Fiscal Measures in Response to the COVID-19 Pandemic (by September 2021)

Country	Above-the-line spending % of GDP	Liquidity support % of GDP	Total fiscal stimuli % of GDP
China	4.8	1.3	6.1
Singapore	18.4	4.7	23.1
South Korea	6.4	10.1	16.6

*Source*: IMF Fiscal Monitor (2021)

Please note: The data comprise fiscal measures taken between early 2020 and September 2021. “Above the line” measures refer to additional spending or foregone revenues in response to the pandemic. “Liquidity support” refers to equity injections, loans, asset purchases, dept assumptions or contingent liabilities that will be paid if a triggering event occurs.

Despite all the caveats that need to be made in comparing fiscal data across regimes, this finding appears remarkable as it reflects a significant difference between autocratic China and hybrid Singapore. While China’s party regime clearly and consistently prioritized the protection of lives, safeguarding people’s livelihoods via proactive fiscal measures seems to be a relatively low priority despite ideologically grounded promises of material wealth and a “better life” for the people. On the other hand, the extensive fiscal stimuli mustered by Singapore’s government to stabilize the economy and support people’s livelihoods seems to confirm the paternalistic nature of PAP dominance under Lee, whose top priority appears to be to maintain the high levels of material wealth accrued by Singapore’s citizens over the course of decades.

To complement the findings on the livelihood variable, the change of real GDP growth rates over time gives additional clues. Over the first two years of the pandemic, the three cases demonstrate a similar pattern. After painful economic slowdowns in 2020 (from 6 to 2.2% in China; 1.1% to −4.1% in Singapore; 2.2% to −0.7% in South Korea), growth rates bounced back to exceed pre-pandemic levels in 2021. The picture changed massively with the advent of the Omicron wave in late 2021, though. As the quarterly rates for the first half of 2022 show, real GDP growth fell significantly to low positive rates in all three countries. While Singapore and South Korea recorded slight improvements in the second quarter, however, China’s real GDP tumbled to a negative growth rate of −2.7% to reach the worst quarterly figure since the outbreak of the pandemic (Table 4). As these data suggest, China’s strict adherence to its Zero-COVID strategy even during the Omicron wave has come not only at the immediate expense of individual rights but also, and in a more protracted manner, at that of people’s livelihoods.

**Table 4:** Real GDP Growth Rates in China, Singapore, South Korea (2019 to mid-2022)

Country	2019 (%, YoY)	2020 (%, YoY)	2021 (%, YoY)	2022 (%, YoY)^a^	2022 Q1 (%, QoQ)	2022 Q2 (%, QoQ)
China	6	2.2	8.1	3.2	1.6	−2.7
Singapore	1.1	−4.1	7.6	3.0	0.4	1.5
South Korea	2.2	−0.7	4.1	2.6	0.6	0.7

*Source*: IMF World Economic Outlook, Datamapper (2022)

Please note: Annual growth rates are given on a year-on-year basis (YoY); growth rates for the first and second quarter of 2022 are compared to previous quarters, seasonally adjusted (QoQ).

^a^estimates

Overall, we find a clear correlation between regime type and responsiveness, that is, the ability to adapt pandemic response strategies to a changing environment. In China, the early commitment to a zero-tolerance approach has created a path dependency on rigorous lockdowns, economic insulation, and nationalist entrenchment. Given the lock-in effects of a party ideology that regards Zero-COVID as proof of the socialist system’s superiority over Western liberal democracy, an exit from national-crisis mode anytime soon appears unlikely. The economic and human costs caused by the country’s unprecedented restrictions—some speak of a “collective psychological trauma” (Cai 2022)—have been increasing tremendously with the spread of the Omicron variant.

The hybrid regime of Singapore, which also initially opted for a zero-tolerance approach, had to learn a painful lesson when COVID-19 spread among migrant workers in March and April 2020. Thanks to strong state capacities and fiscal power, the government was able to overcome this blind spot within a few weeks by introducing a partial lockdown. Cautious attempts since spring 2021 to reopen an economy highly dependent on its international connections, however, have had to be suspended various times in the face of new coronavirus variants, resulting in a staggered and drawn-out transition to Living with COVID (Chong 2022).

South Korea’s democratically elected government never opted for a Zero-Covid approach. Instead, it has been able to modify its pandemic response all along, reacting to changing circumstances in proportionate ways. Governance structures evolved over the virus outbreak’s first three months to facilitate the coordination of countermeasures across ministries as well as between national and local governments, with leadership shifting upward over time. Strategic adjustment has also been visible in the field of economic-response measures, with increasing efforts to steer recovery funds toward the country’s digital and green transition. South Korea’s transition to Living with COVID can be regarded as relatively smooth while accepting increased losses of life in 2022—still a far cry from COVID-19-related death tolls in the US and Europe, though.

## Conclusion

The comparison of evolving COVID-19 policy responses in China, Singapore, and South Korea has demonstrated the merit of taking a longitudinal perspective. This aids our understanding of how response strategies have been adjusted by relevant actors over the course of the pandemic, and how strategic adaptations have varied across regime types. This dynamic approach represents a significant contribution to the literature, which has hitherto investigated pandemic-response strategies in East Asia mainly from a static “capacity” perspective.

Such a longitudinal perspective allows us to map out the different routes these three countries have taken in their navigation of lives, rights, and livelihoods. While the autocratic Chinese party-state has prioritized the protection of lives through rigorous containment of local outbreaks over everything else, hybrid Singapore and democratic South Korea have repeatedly recalibrated the protection of lives and of livelihoods. In the face of the Delta and Omicron waves, Singapore’s government, wedded to its paternalist promise to secure citizens’ material wealth, suspended the economy’s reopening various times, experiencing a drawn-out transition to Living with COVID. South Korea’s government, the only one among the three cases to consistently respect individual rights and freedoms, acknowledged Omicron as a game changer, shifting from its initial priority of saving lives to that of securing livelihoods in a more targeted manner. Accepting moderate levels of death, its gradual transition to a post-pandemic era has been relatively smooth. From mid-2022, South Korea appears to have become one of the most epidemiologically and economically resilient countries worldwide thanks to its dynamic and successful balancing between the protection of lives, rights, and livelihoods. Among the three cases, its democratic regime features the highest degree of responsiveness in dynamically adjusting to varying challenges over the course of the pandemic. From a longitudinal perspective, this demonstrates the close connection between a regime’s responsiveness on the one hand and the ability to balance conflicting targets on the other.

Given the marked socioeconomic differences between the three country cases discussed, the generalizability of our findings is, of course, limited. However, comparing strategic adaptations across these three polities over time, we still can identify significant lessons about critical regime features that support or hinder such policy adjustments in the longer term. What lessons can European democracies draw from the three cases examined? If a zero-tolerance approach is neither desirable or indeed feasible for open democracies, how to address the next pandemic emerging so that infection rates and death tolls are restricted to moderate levels without extensively encroaching on entrenched rights?

First, epidemics are not necessarily “black swans”—unforeseeable and unlikely events of major impact—but more often “grey rhinos”—foreseeable and likely threats with potentially major effects that are not appropriately rationalized and addressed. As the cases of Singapore and South Korea have shown, governments with sufficient analytical capacities can and do learn from past experience, being alert to and taking precautions against health emergencies. The fact that the German government did not systematically take advice from a “National Pandemic Response Plan” last updated in 2017 (RKI 2017) but rather watched the epidemic spreading from East Asia to Italy and the United Kingdom without raising alert levels is a vivid illustration hereof. Next time around, living in a democracy should not be an excuse again for collective inertia in the face of foreseeable threats given now the COVID-19 experience.

Second, the three case studies suggest that stepping up institutional and production capacities are key to coping with health emergencies. Whether autocratic, hybrid, or democratic, governments in all three cases were able to either fall back on existing public-health facilities, medical staff, and equipment or to mobilize the provision and production of critical resources within short periods of time. Stockpiling critical equipment (as in Singapore) or designating specific health facilities for isolation and exclusive treatment of confirmed cases (as in South Korea) should not be beyond us.

Third, the case of South Korea demonstrates that while democracies have greater latitude to adjust pandemic responses over time, strategic adaptation does not happen naturally. Instead, it needs to be consciously organized to generate productive and viable outcomes. The evolution in COVID-19 governance structures from the ministerial to presidential levels in South Korea is a positive example of a dynamic recalibration of response strategies. While taskforce leadership has remained with national agencies throughout, the revised structures have allowed for flexible coordination with local agencies, political responsiveness, and accountability without preinstalled, autocratic, and top-down command hierarchies.

## Footnotes

1 While the conventionally used democracy indices have unequivocally ranked the Chinese one-party regime as authoritarian and South Korea’s presidential republic as democratic over the past decade (except for the EIU’s Democracy Index ranking of South Korea as “flawed democracy” in some of these years), they offer mixed results for Singapore’s parliamentary republic. Freedom in the World has ranked the city-state as “partly free”; the EIU’s Democracy Index lists it as a “flawed democracy”; V‑Dem has it as an “electoral autocracy” (as distinct from “closed autocracies” such as China). According to Ortmann (2019, p. 173), Singapore has faced “growing pluralist challenges” and gradually transformed from a “hegemonic” into a “competitive authoritarian regime” since 2006. Taking these various rankings and expert assessments together, Singapore has significantly transited away from its autocratic past. The city-state is therefore regarded as a hybrid regime.

2 As of November 2022, more than 200 million people were under lockdown. After a high-profile protest staged in Beijing against Xi and his Zero-COVID policy just before the Chinese Communist Party’’s 20th National Congress in mid-October, protest activities have spread across the country (Watanabe 2022).
